# Establishment and validation of a redox-related long non-coding RNAs prognostic signature in head and neck squamous cell carcinoma

**DOI:** 10.1038/s41598-022-26490-7

**Published:** 2022-12-21

**Authors:** Kaitai Liu, Tianyi Huang, Hui Zhang, Hongxia Deng, Ming Tang

**Affiliations:** 1 Department of Radiation Oncology, The Lihuili Hospital, Ningbo Medical Center, Ningbo, Zhejiang China; 2 Department of Otorhinolaryngology Head and Neck Surgery, The Lihuili Hospital, Ningbo Medical Center, Ningbo, Zhejiang China; 3 Department of Otorhinolaryngology Head and Neck Surgery, Ningbo Women and Children’s Hospital, Ningbo, Zhejiang China

**Keywords:** Head and neck cancer, Functional clustering

## Abstract

Reduction and oxidation (redox) reactions occur in living organisms as part of normal cellular metabolism. Here, we established a novel redox-related long non-coding RNAs (rrlncRNAs) signature to predict the prognosis and therapeutic response in Head and neck squamous cell carcinoma (HNSCC). The expression profile and clinical information were obtained from the TCGA project. In total, 10 differently expressed rrlncRNAs associated with prognosis were identified and involved in a prognostic risk score signature by the least absolute shrinkage and selection operator penalized Cox analysis. The area under the receiver operating characteristic curves of the survival rates predicted by the rrlncRNAs signature over one, two, and three years were found to be 0.651, 0.670, and 0.679. Following the completion of the Kaplan–Meier survival study, we discovered that the lower-risk cohort exhibited a much longer overall survival period in contrast with the higher-risk cohort. Univariate and multivariable Cox regression analyses demonstrated that the risk score independently served as a significant predictive factor. GO annotation and KEGG pathway analyses illustrated that the rrlncRNAs signature was strongly associated with immune-related functions as well as signaling pathways. The tumor-infiltrating immune cells, tumor microenvironment, immune-related functions, HLA gene family expression, immune checkpoint genes expression, and somatic variants differed substantially between the low- and high-risk cohorts. Moreover, patients in low-risk group were predicted to present a favorable immunotherapy responsiveness, while in contrast, the high-risk group patients might have a stronger sensitivity to “docetaxel”. According to our findings, the rrlncRNAs signature showed an excellent prognosis predictive value and might indicate therapeutic response to immunotherapy in HNSCC.

## Introduction

Head and neck squamous cell carcinoma (HNSCC) is ranked as the 6th most prevalent malignant tumor on a global scale and the incidence is still continuously increasing^1,2^. Risk factors of the development of HNSCCs include infection with human papillomavirus (HPV), exposure to environmental pollutants, alcohol abuse, and tobacco smoking^3,4^. At present, the most commonly utilized therapeutic approaches for HNSCC are chemotherapy, radiotherapy, and surgery. In addition, immunotherapy, as an emerging strategy for cancer treatment, has also been well represented in the treatment of HNSCC^5–7^. According to studies, pembrolizumab monotherapy is a suitable first-line treatment for patients with PD-L1-positive recurrent or metastatic HNSCC and pembrolizumab combined with chemotherapy is a suitable first-line treatment for recurrent or metastatic HNSCC patients^8^. Nivolumab therapy resulted in prolonged overall survival among patients with platinum-refractory, recurrent HNSCC^9^. In the last 3 decades, the rate of survival for HNSCC patients has increased to a certain extent as a result of advancements in medicine along with breakthroughs in molecular biology^10^. However, due to the asymptomatic nature of early lesions and the risk of recurrence and metastasis, the five-year rate of survival of HNSCC patients is still low at an average of less than 50%^11,12^. Thus, it is of significant clinical importance to develop biomarkers related to the prognosis of HNSCC.

Reduction and oxidation (redox) reactions occur in living organisms as part of normal cellular metabolism, reactive oxygen species (ROS) are generated by these redox events physiologically or pathologically^13^. A redundancy of ROS can induce oxidative damage of macromolecules including proteins, lipids, and DNA, then further enhance the incidence as well as the development of tumors in multistage^14^. Over the past few years, research reports have explored the function played by redox-related products in different cancers and demonstrated the promising value for predicting the prognosis of patients. It was observed that in advanced cervical cancer, the antioxidant levels were significantly declined, while the levels of lipid peroxidation were remarkably increased compared to healthy controls^15^. The researchers also suggested the evaluation of oxidant and antioxidant levels could help to anticipate the treatment responsiveness of advanced cervix cancer to neoadjuvant chemoradiation^16^. By measuring the levels of superoxide dismutase (SOD) activity, nitric oxide (NO), glutathione (GSH), and lipid peroxidation products (LPO) in blood, we discovered that there was an increase in oxidative stress and a simultaneous decrease in antioxidant enzyme activity with the advance in the lung cancer stages^17^. Xiang et.al. demonstrated that selenite induces cell death and apoptosis by the production of superoxide and altering intracellular redox state in human prostate cancer cells^18^. Long non-coding RNAs (lncRNAs) are a subset of RNAs with a length exceeding 200 nucleotides, often overlapping with, or interspersed between multiple coding and non-coding transcripts^19^. It has been revealed that lncRNAs were involved in modulating gene expression via transcription processing, post-transcriptional processing, as well as chromatin alteration^20^. Previous researchers have established some lncRNA signatures, which have excellent prognostic prediction values in various cancers^21^. The reported signatures included lncRNAs with specific functions, such as ferroptosis-related lncRNAs, autophagy-related lncRNAs, some differentially expressed lncRNAs, and immune-related lncRNAs^22^. Lately, a promising signature comprised 9 redox-related lncRNAs in renal clear cell carcinoma was reported, which had the area under ROC curves (AUCs) demonstrating the prediction capacity for survival rates over one, three, and five years whose values were found to be 0.780, 0.746, and 0.764^23^. However, there are no redox-related lncRNAs signatures that have been reported for prognostic prediction in HNSCC so far.

In the present research, we established an innovative ten-redox-related lncRNAs prognostic signature in HNSCC. Furthermore, we investigated the relationship between the signature and clinicopathological characteristics, tumor immune landscape, and somatic variants. Moreover, the predictive values in prognosis and response to immunotherapy and chemotherapy were assessed.

## Materials and methods

### Data resource

**Figure 1:**
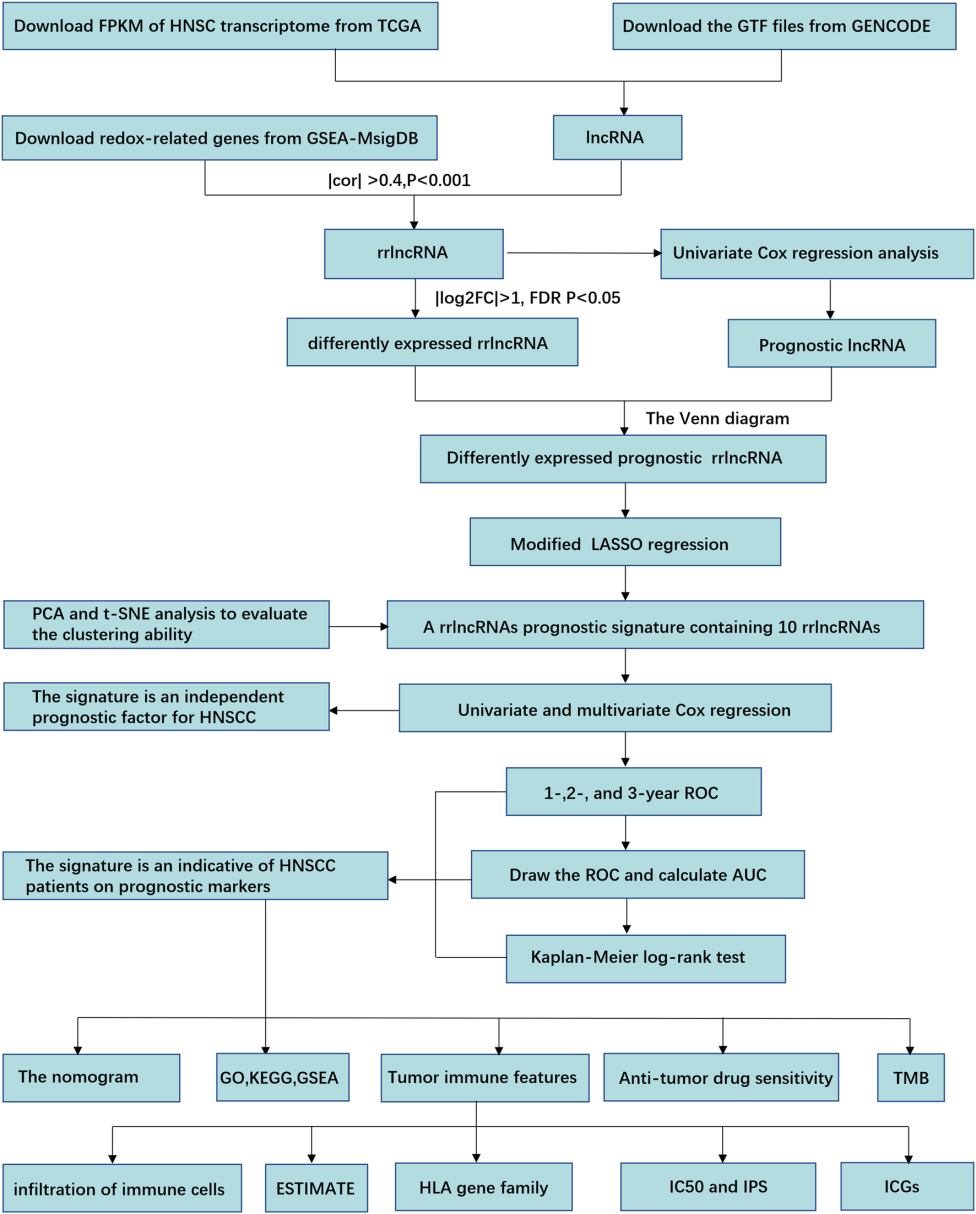
The flowchart of the present research.

The Genomic Data Commons (GDC) Data Portal (https://portal.gdc.cancer.gov) was utilized to acquire gene transcript data from high-throughput sequencing (HTSeq) with normalization in Fragments Per Kilobase of transcript per Million mapped reads (FPKM) as well as simple nucleotide variation data of TCGA-HNSC project. Moreover, clinicopathological and survival data were obtained. By utilizing the GENCODE human website (https://www.gencodegenes.org/human/), we acquired the GTF file of lncRNA annotation (version GRCh38.p13) used in the present research. The redox-related genes (rrGenes) were retrieved from the website of GSEA-MSigDB (https://www.gsea-msigdb.org/gsea/msigdb) according to “redox” as the keyword. The Pearson correlation coefficient was utilized to identify the redox-related lncRNAs having correlation coefficients more than 0.4 and P less than 0.001. Then, we determined that |log2FC|> 1 and false discovery rate (FDR) *P *< 0.05 were the significant criteria for the definition of differently expressed lncRNAs. By means of univariate Cox regression analysis, we determined the prognostic significance of lncRNAs (*P *< 0.05). The Venn diagram was performed to overlap the lncRNAs in the differently expressed cluster and prognostic cluster. Given the fact that no personally identifiable information was utilized in the present research, the Institutional Review Board of the Ningbo Women and Children’s Hospital approved the exemption from the need for ethical clearance for the present research. The study flow was showed in Fig. 1.

### Establishment of a redox-related lncRNAs prognostic signature

The least absolute shrinkage and selection operator (LASSO) penalized Cox regression analyses were utilized to verify and build a redox-related lncRNAs prognostic signature. The risk score of each patient was computed utilizing the equation as follows: Risk score = Coefficient lncRNA^1^ × Expression lncRNA^1^ + Coefficient lncRNA^2^ × Expression lncRNA^2^ + Coefficient lncRNA^3^ × Expression lncRNA^3^ + …… + Coefficient lncRNA^n^ × Expression lncRNA^n^. The patients were divided into low- and high-risk cohorts according to their median risk value. The t-SNE and PCA algorithms were employed to evaluate the clustering ability of this risk score signature. To anticipate the prognosis of HNSCC patients, the one-, three-, and five-year ROC curves of signature were constructed, and the results were compared with standard clinicopathological criteria. Subsequently, we utilized the decision-curve analysis (DCA) plot to assess the normalized overall advantage of the signature and performed a comparison between its benefits and the benefits of other clinical parameters. The Kaplan–Meier method was utilized to create survival curves for various risk cohorts, followed by a comparison. A further investigation was carried out to determine if the rrlncRNAs signature independently served as a prognostic indicator in HNSCC patients utilizing univariate and multivariable Cox regression models. Kaplan–Meier survival curves of subcohort analyses according to age (< 60, ≥ 60), gender (female, male), T stage (T1-2, T3-4), histologic grade (G1-2, G3), lymphatic metastasis (N0, N1-3), and clinical stage (stage I-II, stage III-IV) were severally generated.

### Correlation analysis of the rrlncRNAs signature with clinical variables and establishment of a nomogram

In the present research, we utilized the chi-squared test to delve into the relationship between the clinicopathological characteristics and risk score, which was represented as a heatmap. The *rms* package of the R software was utilized to construct the nomogram that integrated the risk score and clinical and pathological features for evaluating the HNSCC patient's survival rates over one, three, and five years (https://CRAN.R-project.org/package=rms). ROC curves of the nomogram were conducted to evaluate the predictive ability in anticipating the OS rates over one, three, and five years, and the calibration chart was drawn to examine the accuracy of the nomogram.

### Function enrichment analysis

Enrichment analyses on the basis of differentially expressed genes (DEGs) between low and high-risk cohorts were conducted to explore the underlying functional pathways. The threshold value of significantly differential expression was set at FDR *P *< 0.05 and |log2FC|≥ 1. Gene ontology (GO) function analyses and Kyoto Encyclopedia of Genes and Genomes (KEGG) analysis were performed based on DEGs^24,25^. Gene set enrichment analysis (GSEA, version 4.1.0) based on the annotated gene set file (c2.cp.kegg.v7.0.symbols.gmt) was executed to investigate the potential signaling pathways between low and high-risk cohorts in HNSCC.

### Tumor immune feature

Several acknowledged algorithms, such as EPIC, MCPcounter, quanTIseq, CIBERSORT-ABS, xCELL, CIBERSORT, and TIMER were employed to evaluate the discrepancies of tumor-infiltrating immune cells (TIICs) between high- and low-risk cohorts. ESTIMATE algorithm is a method for obtaining ESTIMATE, stromal, and immune scores based on the expression of related molecular biomarkers in immune and stromal cells, which was applied for assessing the tumor microenvironment (TME) disparities in high and low-risk cohorts^26^. Furthermore, single-sample gene set enrichment analysis (ssGSEA) was further used for exploring the immune-related function differences between low and high-risk cohorts. Moreover, the expression divergences of the human leukocyte antigen (HLA) gene family and common immune checkpoint genes (ICGs) retrieved from previous literature^27^ in different risk cohorts were also assessed*.*

### Response prediction to chemotherapy and immunotherapy

To examine the clinical application prospect of the rrlncRNAs signature, we investigated the prediction power of patient prognosis in responding to chemotherapy and immunotherapy in HNSCC. The 50% inhibiting concentration (IC50) values of 4 conventional chemotherapeutic drugs, including “docetaxel”, “cisplatin”, “gemcitabine”, and “pacitaxel” between high and low-risk cohorts, were inferred by utilizing the pRRophetic algorithm. Besides, immunophenoscore (IPS) was calculated to anticipate the potential clinical immunotherapy responsiveness in patients with HNSCC. IPS analysis was a bioinformatics method to quantitatively score the tumor immunogenicity range from 0 to 10, which has been verified for predicting the clinical response to treatment of immune checkpoint inhibitors (ICIs)^28^. In principle, a lower IC50 value and higher IPS value portend a better sensitivity to chemotherapy and immunotherapy, respectively.

### Association between the somatic variants and risk score signature

We identified the topmost twenty commonly mutated genes of HNSCC samples, followed by mutation frequency comparison between high and low-risk cohorts. To calculate tumor mutation burden (TMB), we examined the quantity of somatic, coding, indel, and base substitutions per megabase (Mb) of the genome for subsequent analyses. The TMB score of each sample was calculated by dividing the overall number of mutations counted by the size of the exome used in the calculation process. The size of the exome was estimated to be 38 megabases (Mb)^29^. Furthermore, the association between TMB levels and OS time in HNSCC patients was examined in the present research. Moreover, the prognostic significance of the rrlncRNAs signature was assessed in the low and high TMB subcohorts.

### Statistical analysis

The GESA and HTSeq FPKM data preparation materials were extracted and structured utilizing the Perl software (version: 5.32). The analyses were carried out utilizing the R software (R: A language and environment for statistical computing. R Foundation for Statistical Computing, Vienna, Austria; version: 4.0.3, https://www.R-project.org/) with specialized packages. Statistical significance was determined when the *p-*value was less than 0.05.

### Ethics statement

TCGA is a publicly accessible database. Ethical permission has been acquired for the patients in the databases. Users may get relevant information for free and use it to conduct research as well as publish pertinent publications. Due to the fact that the present research used open-source data, there are no ethical concerns or potentially conflicting interests. The Lihuili Hospital's Institutional Review Board granted its exemption from ethical clearance.

## Results

### Data characteristics

**Figure 2:**
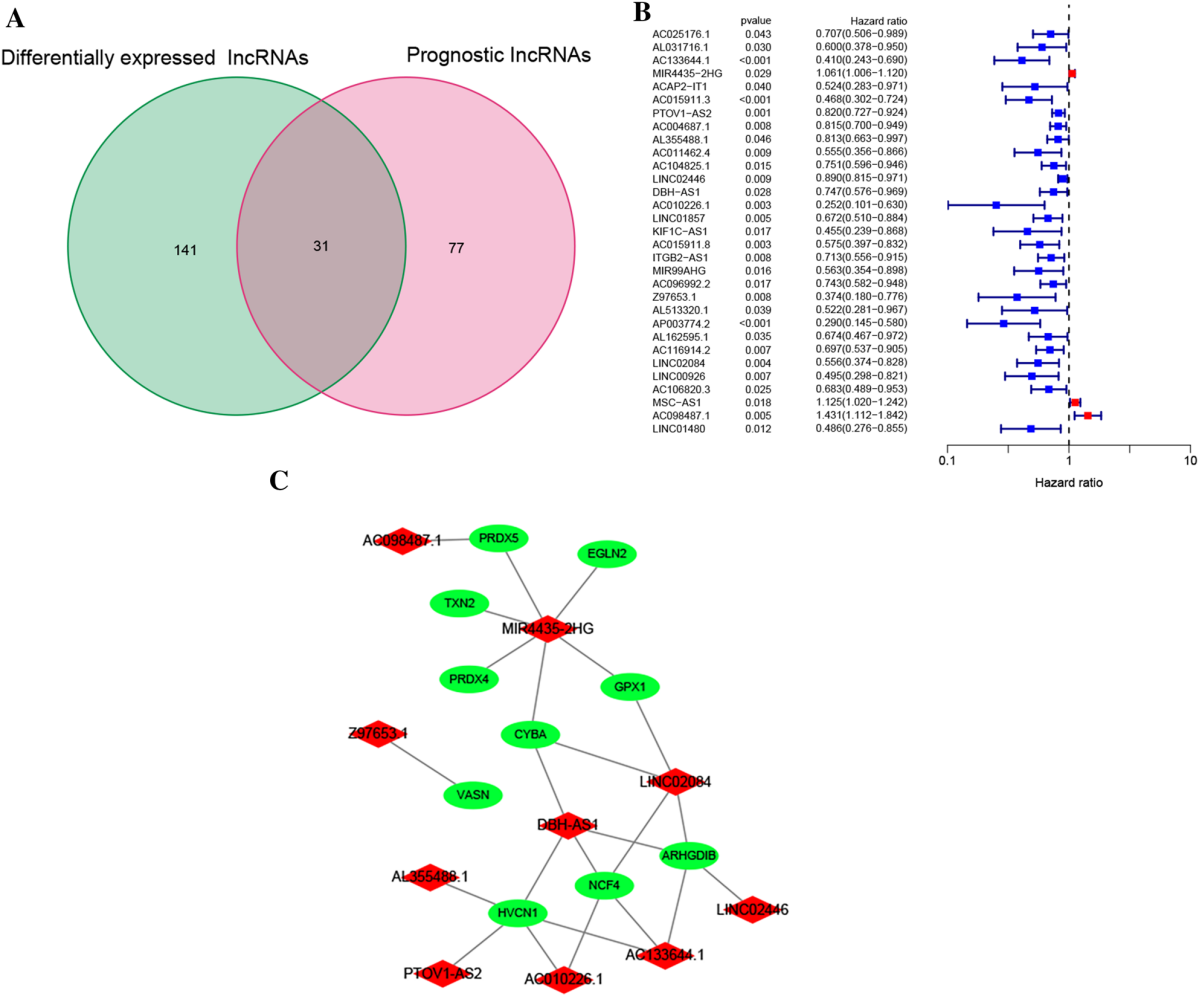
Identification of the rrlncRNAs. (**A**) The 31 overlapped rrlncRNAs extracted from the 172 differentially expressed rrlncRNAs and 108 prognostic rrlncRNAs. (**B**) Univariate Cox analysis of 31 rrlncRNAs in HNSCC. (**C**) The relationship network between identified rrlncRNAs and relevantly co-expressive rrGenes.

The expression profile of 499 HNSCC tissues as well as 44 adjoining normal tissues was included in the present research. Table 1 depicts the patients’ clinical variables, such as gender, age, histologic grade, T stage, lymphatic metastasis, clinical stage, and survival status. In total, 55 redox-related genes (rrGenes) were downloaded and included in the analysis (Table S1). In all, 636 lncRNAs were identified as redox-related lncRNAs (rrlncRNAs) (Table S2). Following with identification of 172 differentially expressed rrlncRNAs and 108 prognostic rrlncRNAs, 31 overlapped rrlncRNAs in the two clusters were extracted, which were used for further analyses (Fig. 2A, B) (Table S3).

**Table 1:** Clinical features of the patients with HNSCC.

Characteristics	Number of patients	Percent
**Gender**		
Female	133	26.65%
Male	366	73.35%
**Age**		
< = 60	279	55.91%
> 60	220	44.09%
**Histologic grade**		
G1-2	359	71.94%
G3	121	24.25%
Unknown	19	3.81%
**T Stage**		
T1-2	177	35.47%
T3-4	267	53.51%
Unknown	55	11.02%
**Lymphatic metastasis**		
N0	170	34.07%
N1-3	236	47.29%
Unknown	93	18.64%
**Clinical stage**		
Stage I-II	94	18.84%
Stage III-IV	337	67.54%
Unknown	68	13.63%
**Survival status**		
Dead	217	43.49%
Alive	282	56.51%

### Construction of a rrlncRNAs risk score prognostic signature

**Figure 3:**
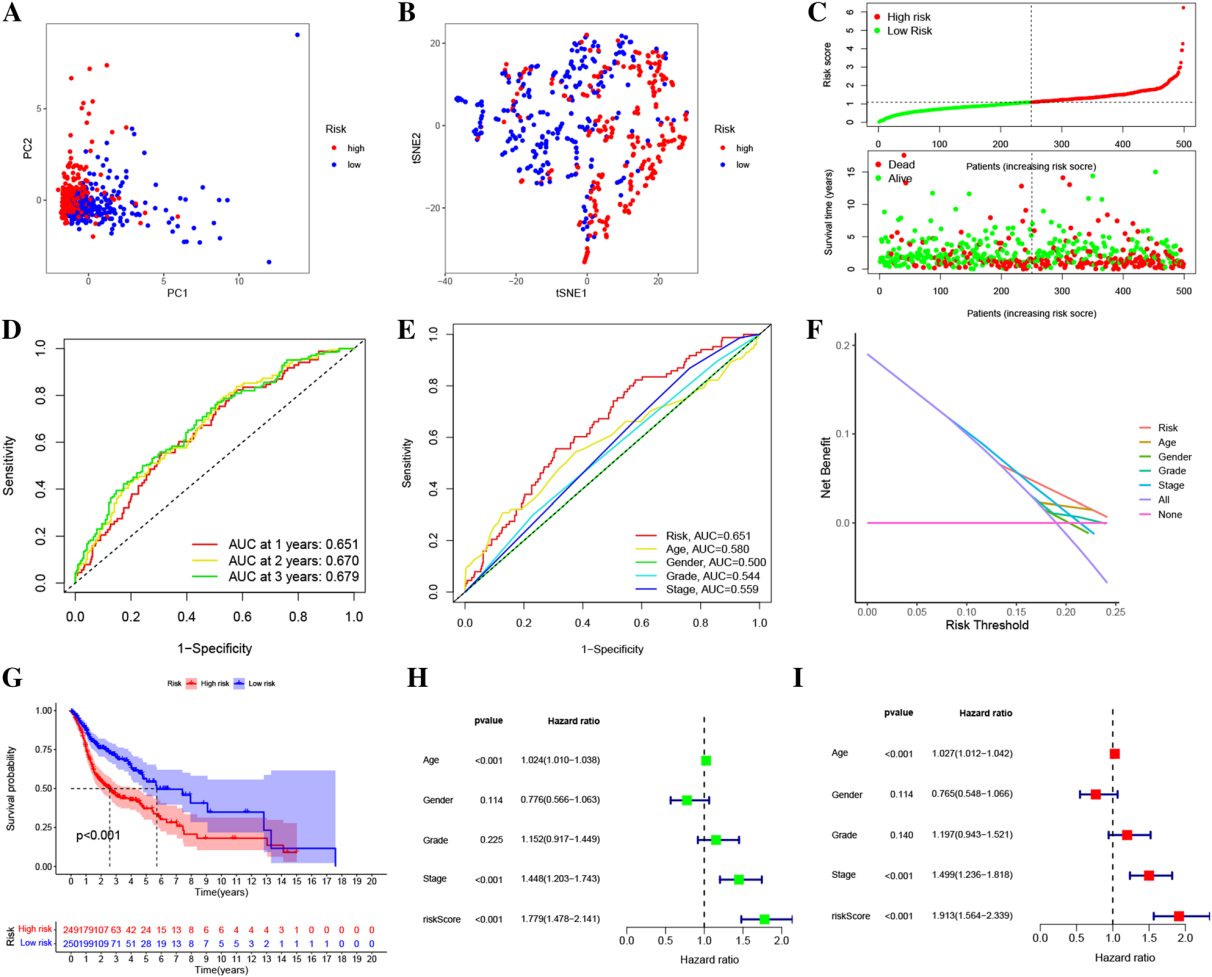
Redox-related lncRNAs prognostic signature constructed using TCGA. (**A**) The PCA plot. (**B**) The t-SNE plot. (**C**) Plot of risk survival status (**D**) The AUC of the signature for predicting the survival rates of HNSCC patients over one, two, and three years. (**E**) The AUC values of the risk factors. (**F**) The DCA of the risk factors. (**G**) Survival analysis using the Kaplan–Meier method for low and high cohorts. (**H**) Univariate Cox analysis of the signature. (**I**) Multivariate Cox analyses of the signature.

According to the least absolute shrinkage and selection operator (LASSO) penalized Cox analysis, 10 out of 31 rrlncRNAs (AC133644.1, MIR4435-2HG, PTOV1-AS2, AL355488.1, LINC02446, DBH-AS1, AC010226.1, Z97653.1, LINC02084, AC098487.1) were identified and used to construct a risk score prognostic signature (Table S4). After that, the relationship network between identified rrlncRNAs and relevantly co-expressive rrGenes (*PRDX5*, *EGLN2*, *TXN2*, *PRDX4*, *CYBA*, *GPX1*, *VASN*, *HVCN1*, *NCF4*, *ARHGDIB*) was constructed (Fig. 2C). The results of PCA (Fig. 3A) and t-SNE (Fig. 3B) analyses showed a favorable clustering power of this rrlncRNAs prognostic signature in HNSCC. Using the risk assessment model for survival status predictions, it was discovered that an increment in the risk score was associated with a substantial rise in the number of patients who died (Fig. 3C). The area under the ROC curves (AUCs) of the risk score signature for survival rates over one, two, and three years were found to be 0.651, 0.670, and 0.679 (Fig. 3D). The AUC value of our signature was positively superior to age (0.580), gender (0.500), grade (0.544), and stage (0.559) in predicting the survival of HNSCC (Fig. 3E). The result of a decision-curve analysis (DCA) plot illustrated that the risk score signature had a most remarkable standardized net benefit compared to other clinical factors, such as stage, gender, grade, and age (Fig. 3F). According to the findings from the Kaplan–Meier survival analysis, the low-risk cohort exhibited a considerably longer overall survival time as opposed to the high-risk cohort (*P *< 0.001, Fig. 3G). These findings revealed that the established rrlncRNAs risk score prognostic signature had a promising clinical prediction efficiency in patients with HNSCC. Moreover, the rrlncRNAs risk score signature was confirmed to independently function as a prognostic marker with the aid of univariate (Fig. 3H) and multivariate Cox regression analyses (Fig. 3I).

### Correlations between the risk score signature and clinical characteristics

**Figure 4:**
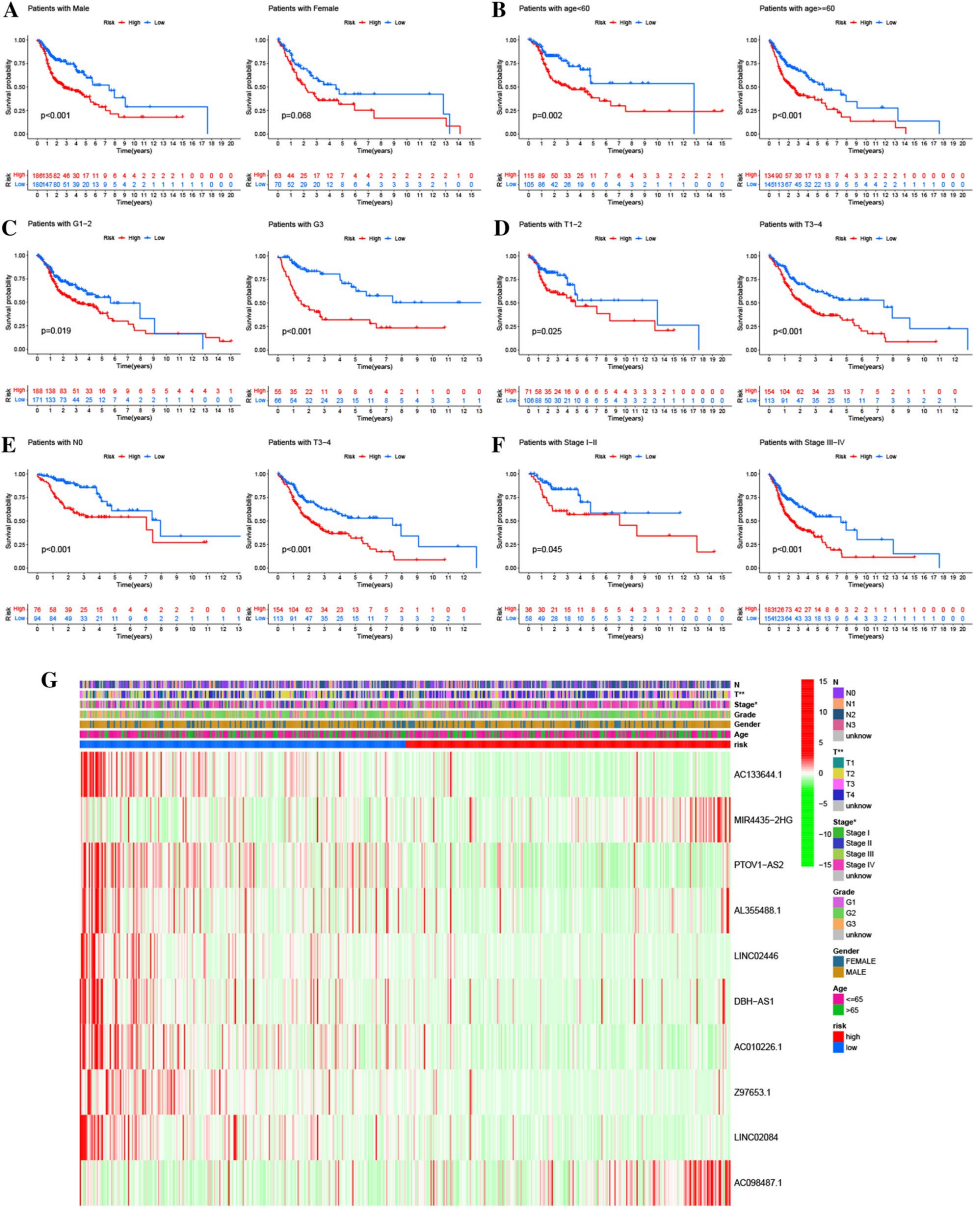
Survival curves of different risk scores in subcohorts. (**A**) Gender, (**B**) Age, (**C**) Histologic grade, (**D**) T stage, (**E**) Lymphatic metastasis, (**F**) Clinical stage; the correlation analysis revealed that stage III/IV and T3/4 patients exhibited an elevated risk score in contrast with the stage I/II and T1/2 patients (**G**).

**Figure 5:**
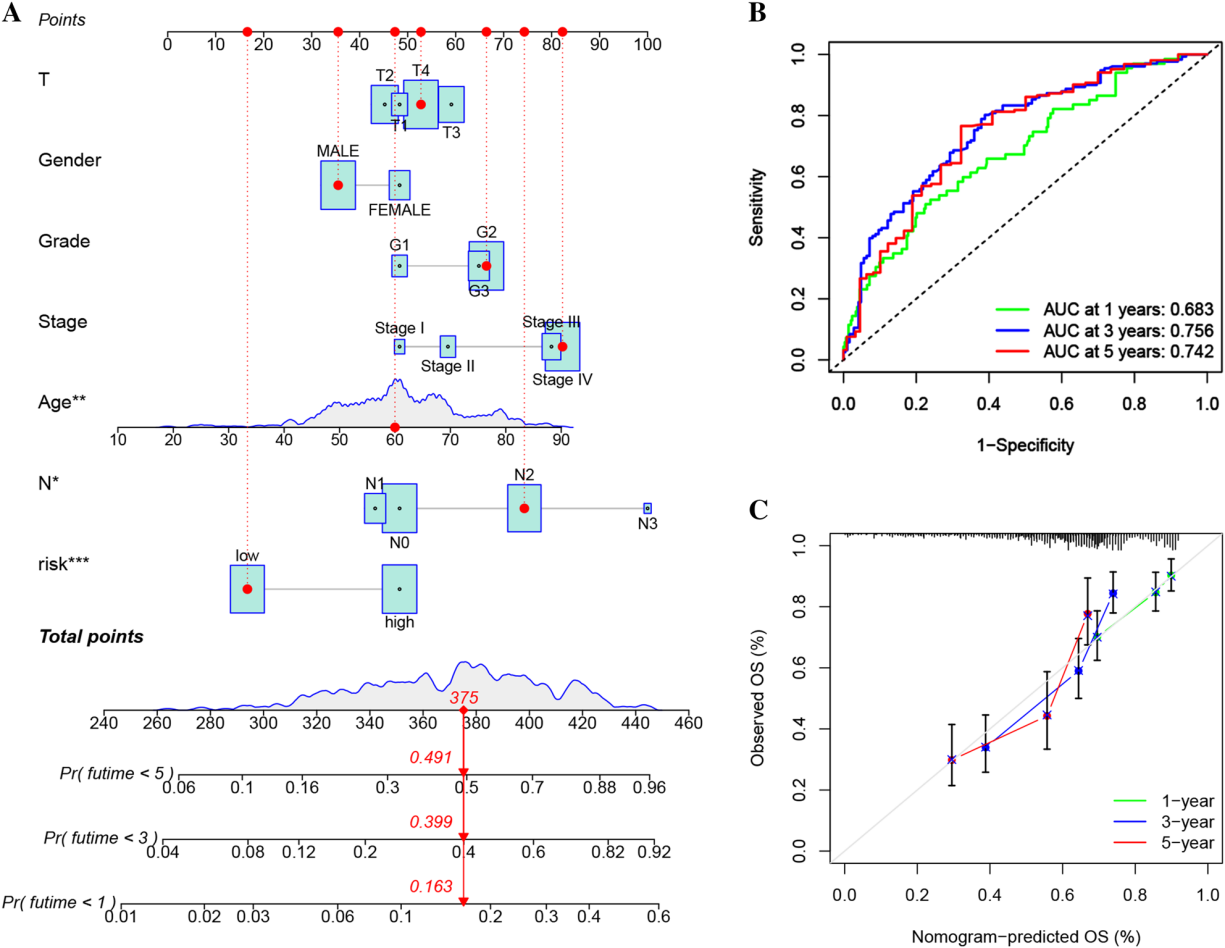
(**A**) A prognostic nomogram for HNSCC patients. (**B**) ROC curves showing the capability of the nomogram in predicting OS over one, three, and five years. (**C**) Calibration plot of the nomogram in forecasting the OS over one-, three- and five-year.

The subgroup analyses of the prognostic prediction effect illustrated that the low-risk cohort had longer OS time as opposed to the high-risk cohort in all subgroups except for the female group (Fig. 4A–F). The correlation analysis revealed that stage III/IV and T3/4 patients exhibited an elevated risk score in contrast with the stage I/II and T1/2 patients (Fig. 4G). Furthermore, a nomogram that incorporated the risk score together with common prognostic clinicopathological factors (gender, N stage, T stage, histologic grade, age, and clinical stage) was successfully generated for estimating the 1-, 3-, 5-year survival probability (Fig. 5A). The AUC of the nomogram for anticipating the OS rates over one, three, and five years were found to be 0.683, 0.756, and 0.742 (Fig. 5B). The calibration plots for OS over one, three, and five years indicated the nomogram showed a promising predictive capacity and performance for HNSCC (Fig. 5C).

### Possible mechanism that underlies the effects of prognostic rrlncRNAs signature

**Figure 6:**
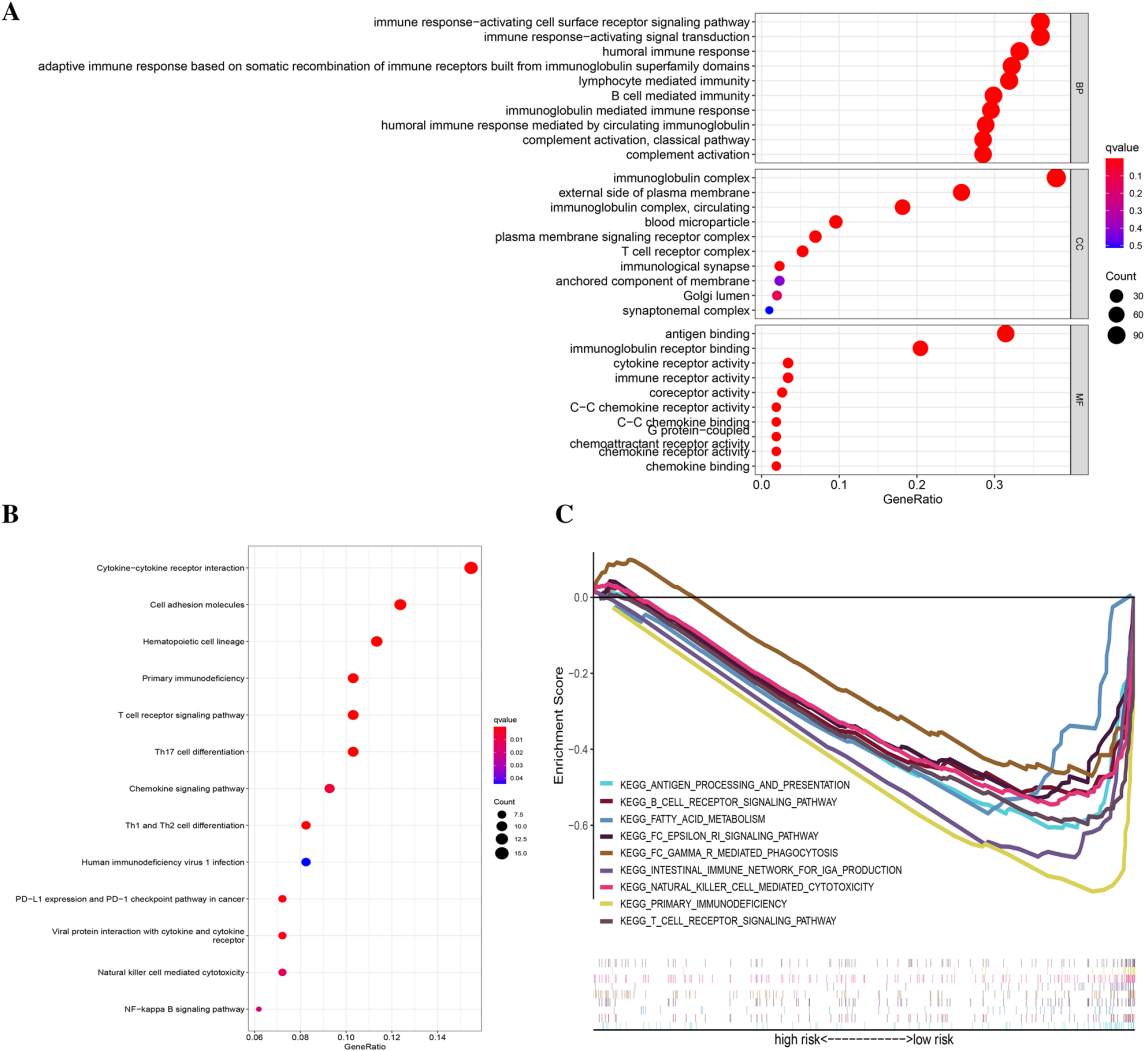
Function enrichment analysis. (**A**) GO analysis of HNSCC patients in low- and high-risk cohorts. (**B**) KEGG analysis of HNSCC patients in low and high-risk cohorts. (**C**) GSEA of HNSCC patients in the low and high-risk cohorts.

The results of GO and KEGG pathway analyses illustrated that the rrlncRNAs signature was strongly correlated with immune-related functions and signaling pathways. The immune-related signaling pathways in biological processes (BP) mainly included “immune response-activating cell surface receptor signaling pathway”, “immune response-activating signal transduction”, “humoral immune response”, “adaptive immune response based on somatic recombination of immune receptors built from immunoglobulin superfamily domains”, “lymphocyte-mediated immunity”, “B cell-mediated immunity”, “immunoglobulin mediated immune response”, “humoral immune response mediated by circulating immunoglobulin”; in molecular functions (MF) included “immunoglobulin complex”, “immunoglobulin complex, circulating”, “T cell receptor complex”, “immunological synapse”; in cellular components (CC) included “antigen-binding”, “immunoglobulin receptor binding”, “immune receptor activity” (Fig. 6A). KEGG pathway analyses revealed additional immune-related signaling pathways, such as “primary immunodeficiency”, “T cell receptor signaling pathway”, “Th17 cell differentiation”, “Th1 and Th2 cell differentiation”, “PD-L1 expression and PD-1 checkpoint pathway in cancer”, “Natural killer cell mediated cytotoxicity”, and so on (Fig. 6B). The findings from GSEA illustrated that the high-risk score cohort exhibited a significant inverse association with several immune-related signaling pathways, which included “antigen processing and presentation”, “B cell receptor signaling pathway”, “FC epsilon RI signaling pathway”, “FC gamma R mediated phagocytosis”, “intestinal immune network for IGA production”, “natural killer cell-mediated cytotoxicity”, “primary immunodeficiency”, and “T cell receptor signaling pathway” (Fig. 6C).

### Association between the tumor immune landscape and rrlncRNAs signature

**Figure 7:**
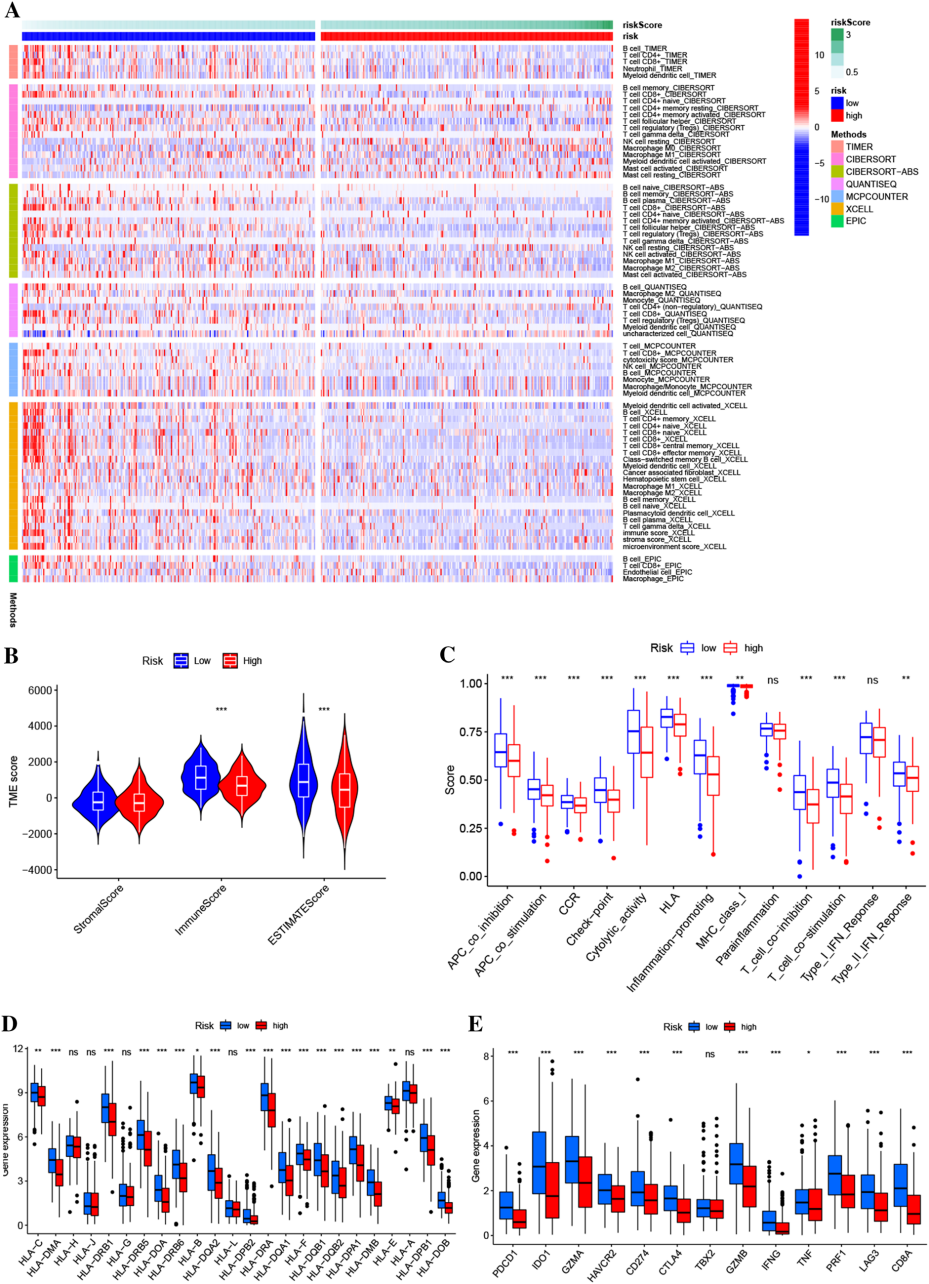
Association between the tumor immune landscape and rrlncRNA signature. (**A**) Heatmap depicting tumor-infiltrating immune cells based on EPIC, CIBERSORT-ABS, XCELL, MCPCOUNTER, QUANTISEQ, CIBERSORT, and TIMER algorithms between low- and high-risk cohorts in HNSCC. (**B**) ESTIMATE analysis between low and high-risk cohorts. (**C**) Immune-related function analyses between low and high-risk cohorts. (**D**) The expression of the HLA gene family between low and high-risk cohorts. (**E**) The expression of immune checkpoints genes between low and high-risk cohorts.

The findings illustrated that the low-risk cohort had a greater proportion of activated immune cells infiltrating which included macrophage, CD4 + T cells, neutrophil, CD8 + T cells, myeloid dendritic cell, B cell, and activated NK cell (Fig. 7A). Furthermore, the ESTIMATE algorithm revealed that the low-risk score cohort exhibited elevated immune and ESTIMATE scores, indicating a higher infiltration of immune cells and attenuated tumor cell purity in the low-risk cohort (Fig. 7B). Immune-related function analyses showed that checkpoint, cytolytic (activity), inflammation-promoting, MHC class I, HLA, T cell co-stimulation, APC co-stimulation, T cell co-inhibition, CCR, APC co-inhibition, and type II INF response were significantly activated in the low-risk cohort (Fig. 7C). Additionally, the expression of the human leukocyte antigen (HLA) gene family was observably elevated in the low-risk score cohort as opposed to the high-risk score cohort, except for *HLA-A*, *HLA-G*, *HLA-J*, *HLA-L*, and *HLA-H* (Fig. 7D). The low-risk cohort also had substantially upmodulated immune checkpoint genes expression, which included *CD8A*, *GZMA*, *PRF1*, *HAVCR2*, *IFNG*, *GAMB*, *CD274*, *TNF*, *IDO1*, *LAG3*, *CTLA4*, and *PDCD1* (Fig. 7E).

### Predictive effects of the rrlncRNAs signature for therapeutic sensitivity

**Figure 8:**
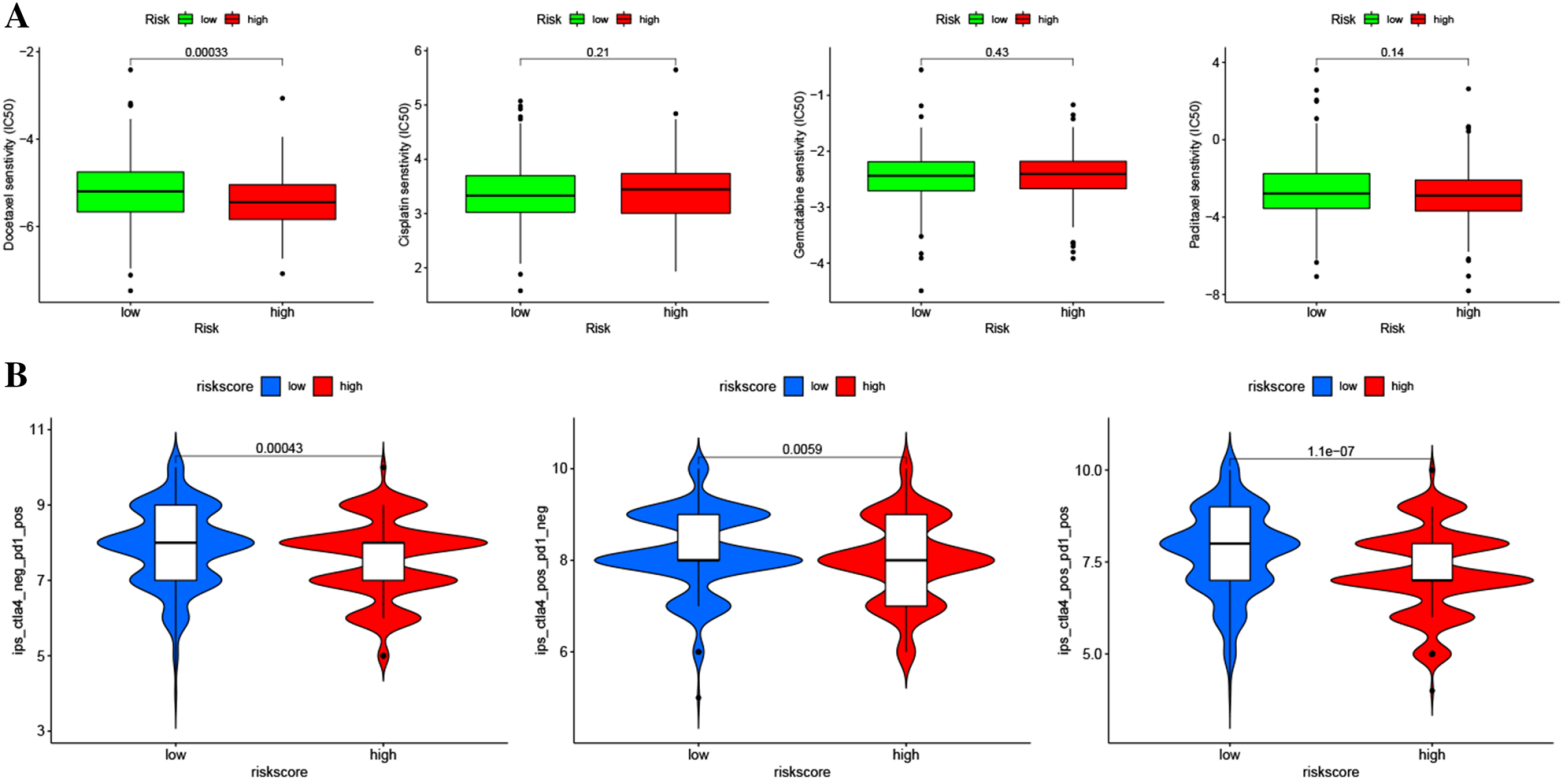
The predictive effects of the rrlncRNAs signature for chemotherapeutic drugs (**A**) and immune checkpoint inhibitors (**B**).

The predictive effect of the rrlncRNAs signature in chemotherapy was evaluated according to the 50% inhibiting concentration (IC50) values of 4 conventional chemotherapeutic drugs, including “docetaxel”, “cisplatin”, “gemcitabine”, and “pacitaxel”. The findings illustrated that the IC50 value of “docetaxel” was considerably elevated in the low-risk cohort as opposed to the high-risk cohort, but there were no substantial differences among the other three drugs. This suggests that the high-risk cohort patients might have a stronger sensitivity to “docetaxel” (Fig. 8A). Besides, the prediction capacity of the rrlncRNAs signature in immune checkpoint inhibitors (ICIs) was evaluated by immunophenoscore (IPS) algorithm. The results demonstrated that the IPS value in the low-risk cohort was considerably elevated in contrast with the high-risk cohort in various ICIs conditions, including “CTLA4_pos_PD1_pos”, “CTLA4_pos_PD1_neg”, and “CTLA4_neg_PD1_pos”. These results suggest a probably favorable immunotherapy responsiveness in the low-risk cohort (Fig. 8B).

### Discrepancy of somatic variants in different risk score cohort

**Figure 9:**
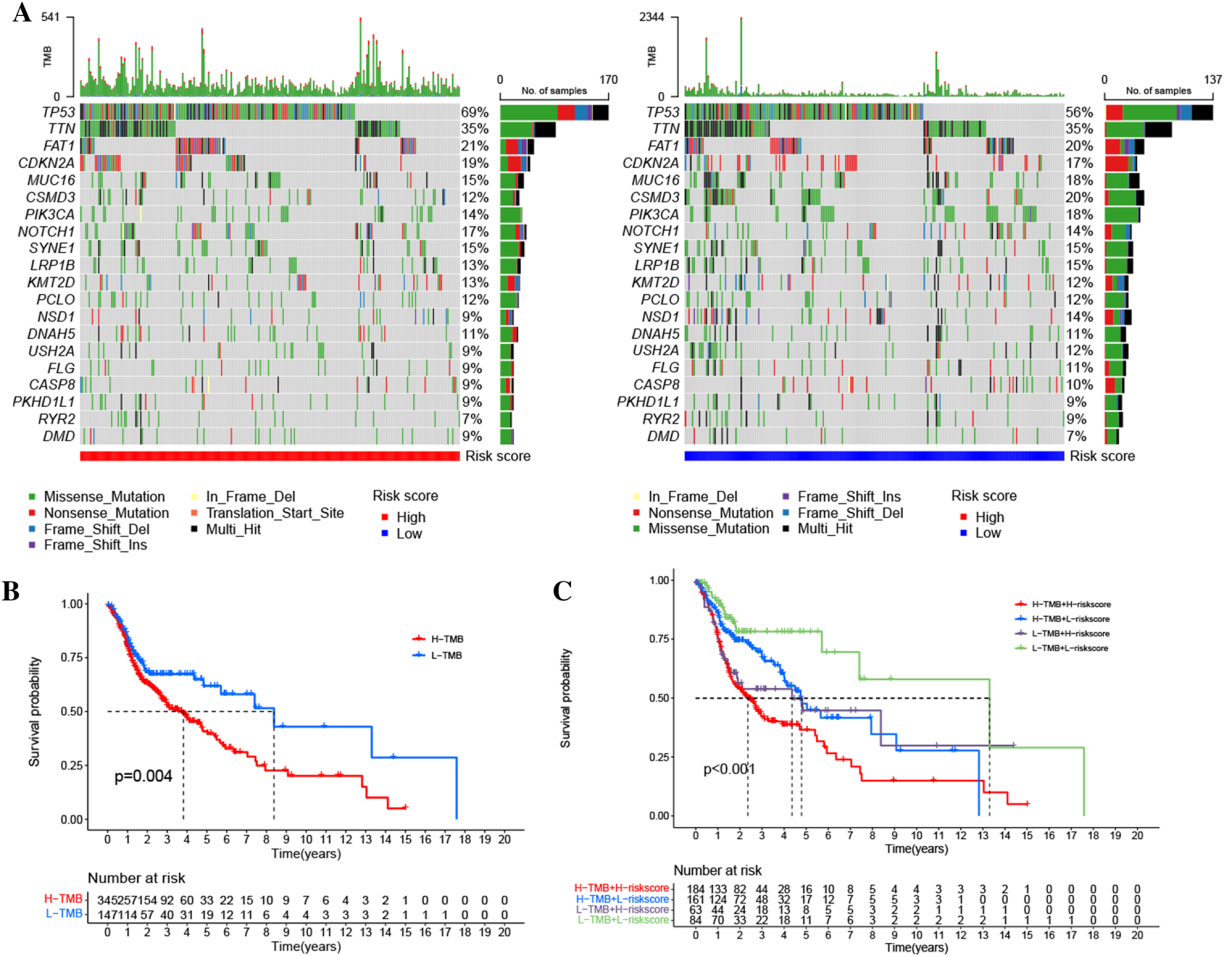
(**A**) The topmost twenty most commonly mutated genes in low and high-risk cohorts. (**B**) Survival curves of low- and high-TMB cohorts. (**C**) Survival curves of high and low-risk cohorts with different TMB levels.

We explored the topmost twenty commonly mutated genes in HNSCC, and the five of the most commonly mutated genes included *FAT1*, *TTN*, *CDKN2A*, *TP53*, and *MUC16*. Furthermore, disparities in mutation rates of these genes were evaluated in low- and high-risk cohorts. These findings demonstrated that *NOTCH1* and *TP53* mutations were highly frequent in the high-risk cohort whereas *CSMD3*, *PIK3CA*, *NSD1*, *USH2A* had obviously increased frequent mutations in the low-risk cohort (Fig. 9A). Moreover, patients exhibiting attenuated TMB levels had significantly longer survival times as opposed to the ones having high TMB (Fig. 9B). We examined the prognostic value of the rrlncRNAs signature in high and low-TMB cohorts. Finally, we discovered that the prognostic signature had similar strong prognostic significance in both the high- and low-TMB cohorts, demonstrating that the TMB status had no influence on the prediction (Fig. 9C).

## Discussion

HNSCC is among the most prevalent contributor to cancer mortality globally. Due to the lack of early screening and the common invasion of regional lymph nodes, most cases are detected at advanced stages, with an unfavorable 5-year survival rate^1,30^. Although HPV detection became an important indicator to guide treatment and predict prognosis in HNSCC^31^, however, due to the tumor heterogeneity, more biomarkers are expected to provide better prognostic and therapeutic efficacy prediction. In HNSCC, accumulation or suppression of ROS occurred at all stages of disease underlying tumorigenesis, development, progression, and response to various therapies on account of alterations in redox metabolism^32^. For the last few years, studies have shown that modulators of redox metabolism were promising strategies for clinical management of HNSCC, including detection and diagnosis, synergizing the efficacy of conventional radiotherapy, chemotherapy, EGFR targeted therapy, and immunotherapy^32,33^. The novel role of specifical lncRNAs signatures as prognosis predictors have been reported in a variety of cancers including breast cancer^34^, laryngeal cancer^35^, lung cancer^36^, colorectal cancer^37^, and HNSCC^38^. Here, we established a redox-related lncRNAs signature as a prognostic biomarker for HNSCC patients, which comprised ten lncRNAs related to redox genes, including AC133644.1, PTOV1-AS2, AL355488.1, LINC02446, DBH-AS1, AC010226.1, Z97653.1, LINC02084, MIR4435-2HG, and AC098487. Research reports have demonstrated the role of lncRNA MIR4435-2HG as an oncogenic lncRNA in various cancers. Dong et al. found an elevated expression of MIR4435-2HG in colorectal cancer (CRC) tissue and promoted CRC growth as well as metastasis via miR-206/YAP1 axis^39^. Gao et al. discovered that MIR4435-2HG is enhanced in gastric cancer cells and that it promotes the proliferation as well as invasiveness of these cells via targeting the miR-138-5p/Sox4 axis^40^. MIR4435-2HG was observed to be markedly upregulated in HNSCC tissues, which could promote cancer progression by modulating the miR-383-5p/RBM3 axis^41^. DBH-AS1 was discovered to be a risk lncRNA associated with an unfavorable prognosis in hepatocellular carcinoma^42^. The rrlncRNA signature showed a promising clinical prediction efficiency and could serve as a prognostic factor in HNSCC. However, the functions of the other eight lncRNAs involved in the signature deserve further exploration.

To investigate the potential biological functions as well as signaling pathways by which our rrlncRNAs signature works in carcinogenicity, GO and KEGG enrichment analyses were performed according to DEGs between the low and high-risk cohorts. The findings demonstrated that the rrlncRNAs signature was substantially enriched in many immune and immune response-related biological functions and signaling pathways. Actually, a recent review summarized the effects of redox signaling pathways on the immune cells' function regulation in cancer^43^. Evidence revealed that ROS and other main cellular antioxidant pathways were closely related to the proliferation, survival, and regulation of the function of T cells, B cells, and macrophages. A review pinpointed operational concepts related to the redox biology network applied to the pathophysiology and therapeutics of solid tumors, including radiotherapy, photodynamic therapy, and anti-tumor immune therapy. The authors draw attention on the consequences of the damage signals delivered by oxidative stress-injured cells to neighboring and distant cells, and emphasize the benefits of therapeutically triggered immunologic cell death in metastatic cancer^44^. David D Roberts reported the role of cellular redox signaling in T cell proliferation and activation^45^. Roberta Vené revealed the vital function of redox remodeling in B-cell activation and differentiation^46^. Xiang Li found that ROS decreased the amount of tumor exo-miR-155 that was taken up by macrophages, resulting in enhanced macrophage infiltration and T cell inactivation characterized by upregulation of PD-L1 in ovarian cancer^47^. According to these results, we speculated that the rrlncRNAs signature might have a promising impact on the tumor immune milieu in HNSCC. Therefore, we further investigated the associations between the established signature and tumor immune landscape in HNSCC. TIICs, a significant constituent of the tumor microenvironment (TME), is shown to perform an integral function in cancer prognosis and response to immune therapy in a variety of tumor types, including lung cancer^48,49^, breast cancer^50^, colorectal cancer^51^ and so on. For patients with HNSCC, research conducted by Zhou et.al. suggested the cell structure of TIICs is remarkably different in HNSCC with or without HPV infection and the variances are crucial for the therapeutic outcome and the development of the tumor^52^. In the present research, our analysis of immune-infiltrated cells enrichment showed a significant discrepancy between low- and high-risk score cohorts, suggesting that the prognosis predictive effect may be correlated with its clustering efficiency in tumor immune features. TME score evaluated by ESTIMATE analysis demonstrated that the low-risk score cohort exhibited elevated immune and ESTIMATE scores, indicating a greater tumor purity in the high-risk cohort. Immune-related function analyses showed that checkpoint, cytolytic (activity), T cell co-inhibition, APC co-inhibition, T cell co-stimulation, inflammation-promoting, HLA, MHC class I, APC co-stimulation, CCR, and type II INF response differed substantially between the high- and low-risk score cohorts.HLA class I might be additionally subdivided into HLA-A, -B, and -C subclasses based on the location of the encoding gene in each of these subclasses. HLA class I molecules are essential for presenting tumor antigens to naïve T cells, whereas HLA class II molecules stimulate the conversion of these naïve T cells into activated T cells by delivering exogenous antigen peptides to CD4 + T cells^53,54^. Actually, according to the findings of numerous research reports, the expression of MHC on tumor cells serves as an effective surrogate indicator for the general immunogenicity of the tumor and also a predictive indicator for immunotherapy response^55^. As shown in the results of two distinct clinical trials, CheckMate 064 and CheckMate 069, attenuated expression of tumor HLA class I molecule (≤ 30%) was linked to an absence of response to ipilimumab, while increased expression of tumor HLA class II molecule (> 1 percent) was associated with greater tumor responsiveness to nivolumab^56^. In the present research, we discovered that the expression of the human leukocyte antigen (HLA) gene family was observably elevated in the low-risk score cohort as opposed to the high-risk score cohort, suggesting the predictive value of the signature in response to immune therapy. Notablely, we found that the patients in high-risk cohort might have a stronger sensitivity to “docetaxel”. Similar studies have shown that the sensibility to “docetaxel” was higher in the high-risk patients in HNSCC based on a hypoxia-related lncRNA model and a cuproptosis-related lncRNA signature^57,58^. Wei Zhang et al. revealed that lncRNA LINC00184 could promote “docetaxel” resistance and immune escape in prostate cancer cells and lead to an increase in PD-L1 expression^59^. We speculate that the lncRNAs involved in our signature might influence the sensitivity of cancer cells to docetaxel by regulating redox reaction, hypoxia, cuproptosis, immune microenvironment and other factors, which is worth further exploration and research.At present, the main mechanism of immunotherapy is focused on the blocking of immune checkpoints. In the present research, we discovered that HNSCC with a low-risk score exhibited significantly upmodulated expression of immune checkpoint genes including *CD8A*, *HAVCR2*, *LAG3*, *IFNG* (*IFNγ*), *GZMB*, *CTLA4*, *TNF*, *CD274*, *PRF1*, *GZMA*, and *PDCD1*. *PDCD1* (*PD1*), *CD274* (*PD-L1*), and *CTLA4* are three already known immune checkpoints, numerous studies have achieved great success in immunotherapy targeting *PD1*/*PDL1* therapy in different solid tumors in the past decade^60^, and other immune checkpoint inhibitors have also made substantial progress. *IDO1* is a new immunological checkpoint target that is defined as a rate-limiting metabolic enzyme responsible for transforming tryptophan into kynurenines. Despite the growing evidence that blocking *IDO1* alone does not produce therapeutic benefit, there is convincing evidence for the use of combined methods to deliver a synergistic advantage to patients^61^. One recent study revealed that as *IDO1* expression level increases, so do other immune checkpoints, including *PD-L2*, *CTLA-4*, *PD-L1*, *PD-1*, and so on. In addition, one research showed the high expression of *IDO1* was induced by IFNγ that inhibited the STAT1 signaling pathway cooperatively, which led to suppressing the process of cell death and activating the dormancy program^62^. *GZMA* and *PRF1* are two key cytolytic effectors, tightly co-expressed, dramatically upregulating upon CD8 + T cell activation. It has been demonstrated that the geometric mean of *PRF1* and *GZMA* expression is correlated with both the neoantigen load and the local immune infiltrates cytolytic activity^63^. *HAVCR2* also named *TIM3*, was expressed by IFNγ-producing CD8 + T cells, CD4 + T cells, as well as other cell types. *TIM3* is a negative immunological checkpoint that is critical in the inhibition of the immune system induced by tumors. Another research discovered that the transcription factor *TIM3* was correlated with the immunosuppressive impact in HNSCC and that targeting *TIM3* may boost anti-tumor immune reaction by attenuating Tregs activity in HNSCC^64^. Tumor necrosis factor (*TNF*), consisting of *TNF-β* and *TNF-α*, is mainly expressed on activated macrophages and lymphocytes. *TNF-α* is also a potent pro-inflammatory cytokine, which plays a critical role in the inflammatory response^65^. Because of its significant prognostic significance, *LAG3* is often recognized as the most important immunotherapy target inferior only to *PD-1*/*PDL-1*. *LAG3* is not only expressed on naïve T cells, as is the case with *CTLA-4* and *PD-1*, but also stimulated on CD8 + T and CD4 + cells in response to antigen presentation. It cooperated with *PD-1* to attenuate antitumor immunity and autoimmunity^66^. Lots of reports revealed the elevated expression of LAG3 and LAG3 + cells infiltration in tumors is correlated with tumor progression, an unfavorable prognosis, as well as adverse clinical outcomes in a variety of human tumors, including colorectal cancer^67^, renal cell carcinoma^68^ as well as HNSCC^69^. Additionally, the IPS analyses also illustrated that the low-risk score cohort exhibited a favorable clinical response to ICIs treatment, which is logically consistent with the above ICGs analyses.

Since TMB was demonstrated to be capable of predicting immune checkpoint blockade therapy efficacy and could be a useful biomarker for the identification of patients that will benefit from immunotherapy across many cancer types^70^, we explored the topmost twenty commonly mutated genes in HNSCC and the difference of mutation frequency in these genes. The results revealed *NOTCH1* and *TP53* mutations were highly frequent in the high-risk cohort while *CSMD3*, *PIK3CA*, *NSD1*, *USH2A* had obviously higher common mutations in the low-risk cohort. *CSMD3* mutation and TME are rarely discussed by researchers, despite the fact that it is one of the most commonly mutated genes in cancers. Several research reports have demonstrated that the *CSMD3* mutation is correlated with favorable survival and one report introduced a case of high-grade serous ovarian cancer with *CSMD3* mutated exhibited a long-term response to anti-PD1 without prior chemotherapy^71^. *NSD1* mutation is also suggested to be a favorable biomarker correlated with favorable survival in HPV negative HNSCC, and its loss-of-function increases sensitivity to cisplatin^72^. Additionally, *PIK3CA*, *TP53*, and *NOTCH1* mutation are well known associated with aberrant activation of oncogenic pathways^73^. Overall, our analysis is consistent with the previous study, except that in our study, *PIK3CA* exhibited highly frequent mutations in the low-risk cohort, on the contrary, the underlying mechanism may need further exploration. Moreover, our result confirmed the prognosis predictive effect of the rrlncRNAs signature in high and low TMB cohorts.

## Conclusion

In conclusion, the ten-redox-related lncRNAs signature showed an excellent prognosis predictive value in HNSCC. Furthermore, the rrlncRNAs signature was associated with tumor immune landscape in HNSCC. Moreover, this signature might indicate a response to chemotherapy and immunotherapy in HNSCC.

## Supplementary Information

Supplementary Information 1.

Supplementary Information 2.

Supplementary Information 3.

Supplementary Information 4.

Supplementary Information 5.

## Data Availability

The datasets generated during the current study are available in the public database for The Cancer Genome Atlas (TCGA) program, which is publicly available at https://portal.gdc.cancer.gov. Correspondence and requests for materials should be addressed to M.T.
