# Supplementary material for: Establishment and validation of a redox-related long non-coding RNAs prognostic signature in head and neck squamous cell carcinoma
Source: Sci Rep. 2022 Dec 21;12:22040. doi: 10.1038/s41598-022-26490-7 (PMC9772388; doi:10.1038/s41598-022-26490-7)
Supplement: Supplementary file 1 — Supplementary Information 1. [file 41598_2022_26490_MOESM1_ESM.pdf]

Supplementary description

supplementary table 1：The 55 redox-related genes (rrGenes) included in the analysis.

supplementary table 2：The list of the 636 redox-related lncRNAs (rrlncRNAs).

supplementary table 3： The 31 overlapped rrlncRNAs in the two clusters of the differentially expressed rrlncRNAs and the prognostic rrlncRNAs.

supplementary table 4： The list of the 10 rrlncRNAs which were identified and used for constructing a risk score prognostic signature.
