# Supplementary material for: Establishment and validation of a redox-related long non-coding RNAs prognostic signature in head and neck squamous cell carcinoma
Source: Sci Rep. 2022 Dec 21;12:22040. doi: 10.1038/s41598-022-26490-7 (PMC9772388; doi:10.1038/s41598-022-26490-7)
Supplement: Supplementary file 2 — Supplementary Information 2. [file 41598_2022_26490_MOESM2_ESM.pdf]

Gene ID
APEX1
CYBA
CYBB
DDIT3
DLD
EGLN2
ERO1A
ERP44
GCLC
GIT1
GPX1
GSR
HVCN1
KRIT1
LPO
MPO
NCF1
NCF2
NCF4
NFE2L2
NNT
NOS1
NOS2
NOS3
NQO1
PRDX1
PRDX2
PRDX3
PRDX4
PRDX5
PRDX6
RAC2
SELENOT
SLC11A1
SLC2A10
TXN
TXN2
TXNDC2
TXNRD1
TXNRD2
TXNRD3
ADH5
ARHGDIB
ARNTL
CLOCK
FKBP1B
GLRX2
NPAS2
RNF7
RYR2
SELENOS
SIRT2
SLC7A11
SMPD3
VASN
