# Supplementary material for: Establishment and validation of a redox-related long non-coding RNAs prognostic signature in head and neck squamous cell carcinoma
Source: Sci Rep. 2022 Dec 21;12:22040. doi: 10.1038/s41598-022-26490-7 (PMC9772388; doi:10.1038/s41598-022-26490-7)
Supplement: Supplementary file 4 — Supplementary Information 4. [file 41598_2022_26490_MOESM4_ESM.pdf]

Gene ID
AC025176.1
AL031716.1
AC133644.1
MIR4435-2HG
ACAP2-IT1
AC015911.3
PTOV1-AS2
AC004687.1
AL355488.1
AC011462.4
AC104825.1
LINC02446
DBH-AS1
AC010226.1
LINC01857
KIF1C-AS1
AC015911.8
ITGB2-AS1
MIR99AHG
AC096992.2
Z97653.1
AL513320.1
AP003774.2
AL162595.1
AC116914.2
LINC02084
LINC00926
AC106820.3
MSC-AS1
AC098487.1
LINC01480
