# Supplementary material for: Establishment and validation of a redox-related long non-coding RNAs prognostic signature in head and neck squamous cell carcinoma
Source: Sci Rep. 2022 Dec 21;12:22040. doi: 10.1038/s41598-022-26490-7 (PMC9772388; doi:10.1038/s41598-022-26490-7)
Supplement: Supplementary file 5 — Supplementary Information 5. [file 41598_2022_26490_MOESM5_ESM.pdf]

id	coef	HR	HR.95L	HR.95H	pvalue
AC133644.	-0.68136	0.505929	0.278518	0.919024	0.025271
`MIR4435-	0.060533	1.062402	0.995848	1.133404	0.066665
`PTOV1-AS	-0.23081	0.793889	0.652812	0.965454	0.02077
AL355488.	0.280866	1.324276	0.99317	1.765767	0.055713
LINC02446	-0.06827	0.934009	0.844578	1.03291	0.183708
`DBH-AS1`	0.563516	1.756839	1.198184	2.575967	0.003903
AC010226.	-0.82556	0.43799	0.152616	1.256981	0.12484
Z97653.1	-0.96362	0.381508	0.183255	0.794236	0.010002
LINC02084	-0.38159	0.682772	0.429894	1.084402	0.105948
AC098487.	0.294819	1.342883	1.012708	1.780704	0.040592
